# Gastroscopy for dyspeptic symptoms in patients <65 years has a low yield of clinically important findings: a retrospective study

**DOI:** 10.1093/jcag/gwae003

**Published:** 2024-02-23

**Authors:** Brooke Maracle, Katelynn Crick, Kerri Novak, Denise Campbell-Scherer, Sander Veldhuyzen van Zanten, Daniel C Sadowski

**Affiliations:** Department of Medicine, University of Calgary, Calgary, AB T2N 1N4, Canada; Office of Lifelong Learning & the Physician Learning Program, Faculty of Medicine and Dentistry, University of Alberta, Edmonton, AB T6G 1C9, Canada; Division of Gastroenterology and Hepatology, Department of Medicine, University of Calgary, Calgary, AB T2N 1N4, Canada; Office of Lifelong Learning & the Physician Learning Program, Faculty of Medicine and Dentistry, University of Alberta, Edmonton, AB T6G 1C9, Canada; Department of Family Medicine, Faculty of Medicine and Dentistry, University of Alberta, Edmonton, AB T6G 2T4, Canada; Digestive Health Strategic Clinical Network, Alberta Health Services, AB, Canada; Division of Gastroenterology, Department of Medicine, University of Alberta, Edmonton, AB T6G 2X8, Canada; Division of Gastroenterology, Department of Medicine, University of Alberta, Edmonton, AB T6G 2X8, Canada

**Keywords:** Dyspepsia, Gastroscopy, Clinical Practice Guidelines

## Abstract

**Background:**

Dyspepsia is a common, generally low-risk gastrointestinal condition. The American College of Gastroenterology and Canadian Association of Gastroenterology recommend avoiding gastroscopy in healthy patients <60 years old. Many dyspeptic patients can be effectively managed in primary care. This study aimed to determine: (1) the proportion of gastroscopies performed for dyspepsia among patients <65 years old with no alarm symptoms or clinically appropriate indications and (2) to determine the frequency of clinically actionable findings and dyspepsia-related healthcare utilization in the year following gastroscopy.

**Methods:**

Outpatient endoscopy reports were sampled and reviewed retrospectively from 2019 to –2021 in Edmonton, Alberta to identify gastroscopies performed for the indication of dyspepsia. Gastroscopies were considered low-risk for significant endoscopic findings if age <65, no alarm symptoms or other concerning indications, and insufficient evidence that first-line treatments and diagnostic approaches had been tried prior to gastroscopy. Clinically important findings were defined as those impacting management, not otherwise identifiable non-invasively.

**Results:**

Of the 358 reviewed gastroscopies for dyspepsia, 293 (81.8%) had no alarm symptoms, and 130 (36.3%) had no alarm symptoms or other appropriate indications. Clinically important findings were identified in 9 (6.9%) of the 130 low-risk cases. In the year following, one patient (1/130) visited the emergency department 3 times for their symptoms and no patients required hospital admission. No malignancies were detected.

**Conclusions:**

Many gastroscopies are performed on patients <65 years old with dyspepsia, even when they lack alarm symptoms or other clinical indications, despite recommendations against this practice and low procedure yield. Strategies to improve the uptake of current guidelines may optimize endoscopy resource utilization.

## Introduction

Dyspepsia is common, with an estimated prevalence of 8% in North America and is defined as a symptom complex of epigastric pain, upper abdominal fullness, early satiety, bloating, and/or nausea.^1–4^ Gastroscopy for dyspepsia has normal endoscopic findings in >75% of patients and is rarely due to esophageal or gastric cancer if no alarm symptoms (sometimes called “red flags”) are present.^5–7^ A recent updated meta-analysis on clinically important endoscopic findings in dyspepsia patients reported normal findings in 67% of Western societies.^8^ Erosive esophagitis was the most common finding at 8.5%–20%.^8^ In the 2003 Canadian CADET Prompt Endoscopy study gastroscopies were reported as abnormal in 58% of patients. Importantly in that study patients had not been on any acid suppressive therapy and endoscopic gastritis was also considered a significant abnormality.^9^

While patients with dyspepsia do not experience a decrease in life expectancy, they do experience reduced quality of life.^10,11^ Patients with dyspepsia represent ~ 2%–5% of primary care visits and account for up to 17% of all referrals to gastroenterologists.^1,12–14^ Choosing Wisely Canada, the Canadian Association of Gastroenterology (CAG) and the American College of Gastroenterology (ACG) currently recommend against gastroscopy in patients <60 years with dyspepsia, unless there are alarm symptoms.^15–17^ Even when alarm symptoms are present, the risk of malignancy is low.^18^ Most patients with dyspepsia can be managed empirically in primary care through lifestyle modifications and trials of anti-secretory medications and/or testing for *Helicobacter pylori*.^5,16,17^ Some patients also benefit from counseling and/or evaluations of mood disorders as the brain–gut interaction can be an important underlying contributing factor.^16,17^

As listed in the American Society of Gastroenterology and Endoscopy (ASGE) and CAG Appropriate Use of GI Endoscopy guideline, >80% of gastroscopies in an endoscopy unit should have appropriate indications.^19,20^ Non-urgent, routine gastroscopies for dyspepsia have long wait times in Canada and are costly to the healthcare system-estimated at $828/gastroscopy excluding the physician fee.^21,22^ The cost to discover a single malignancy in dyspeptic patients <50 years without alarm symptoms has been estimated to cost $82 900 in the United States.^23^

The purpose of this study is to determine the proportion of gastroscopies performed for the investigation of dyspepsia in patients <65 years without alarm symptoms in a Canadian healthcare environment. This information will be used to inform interventions aimed at reducing the performance of low-value gastroscopies for dyspepsia.

## Methods

This study was a retrospective cohort design carried out in the Edmonton Zone (Alberta, Canada). In Alberta, public healthcare is delivered by Alberta Health Services (AHS). The Edmonton Zone (EZ) serves a population of 1 422 000 people and has 7 hospitals where endoscopy services are performed. Using hospital service logs, we identified outpatient gastroscopies for patients aged 18–65 in the EZ. Data were collected from 2 time periods: January 1 to September 30, 2019 (9 months) and January 1 to February 28, 2021 (2 months).

During the first period, 7383 outpatient gastroscopies among patients aged 18–65 were identified. Among these, 1143 had an indication code of dyspepsia and 1510 for “other diagnostic” purposes. From the dyspepsia and “other diagnostic” groups, 400 and 100 cases, respectively, were randomly sampled and screened for inclusions.

In the second period, 741 outpatient gastroscopies were identified in patients aged 18–65 using a new Electronic Medical Record (EMR) at 4 hospital sites. All 741 cases were reviewed for inclusion as indication codes were unavailable in the EMR.

Our inclusion criteria included dyspepsia as the procedure indication, outpatient setting, and ages 18–65. The upper age limit was 65, following the Choosing Wisely recommendation at that time.^15^ We manually reviewed paper endoscopy reports and electronic health records (Epic Verona, WI, USA) to identify dyspepsia cases and collect outcomes data.

For our analysis, a gastroscopy was considered performed for the indication of dyspepsia if the symptoms described included abdominal pain, epigastric pain, upper abdominal pain, dyspepsia, nausea, bloating, and/or epigastric burning.^1–3^ If there were no alarm symptoms or other clinically appropriate indications, gastroscopies were defined as low-risk dyspepsia procedures. Alarm symptoms, or signs, were defined as persistent vomiting, dysphagia, weight loss, anemia, or evidence of gastrointestinal bleeding.^2^ Other clinically appropriate indications included: abnormal diagnostic imaging, abnormal laboratory values such as iron deficiency anemia or positive celiac serology, significant comorbidities including Crohn’s disease, family history of GI cancer, or treatment-resistant *Helicobacter pylori.* These patients were removed from the low-risk dyspepsia cohort.

The Digestive Health Strategic Clinical Network as part of Alberta Health Services (AHS) has created several clinical care pathways including gastroesophageal reflux disease and dyspepsia.^24–27^ The clinical care pathways aim to provide evidence-based guidance to primary care physicians and to optimize management in the medical home, that is, empower the family physician to do more. This may prevent a referral that is not necessary and enhance referral appropriateness for patients with alarm symptoms, non-response to treatment, or persistent symptoms despite pathway adherence.

On chart review, if evidence of adherence to the dyspepsia pathway existed, the indication for the procedure was considered appropriate and removed from the low-risk cohort. Pharmaceutical data was obtained from the Pharmaceutical Information Network. Pathway adherence was defined as those having appropriate lab work, testing for *Helicobacter pylori*, and evidence of a proton pump inhibitor trial completed within the year prior to the gastroscopy. If *Helicobacter pylori* was positive, adherence to the pathway was defined as dispensation of *Helicobacter pylori* treatment (triple/quadruple therapy with antibiotics) and proton pump inhibitors within one year prior to the gastroscopy.^27–30^ All gastroscopy, pathology, and radiology reports were reviewed by 2 gastroenterologists (SVvZ, DS) to determine if the findings were clinically significant. Clinically significant findings were defined as diagnoses impacting medical management including those with immediate consequences, such as peptic ulcer disease, and those with potential for long-term management implications. The same definitions were used in a similar study conducted in Calgary.^30^ Findings were not considered clinically actionable if they could commonly be made noninvasively such as *Helicobacter pylori* infection or celiac disease. The histologic finding of celiac disease was not considered actionable because a tissue transglutaminase (tTg-IgA) test could have preceded the gastroscopy. A positive tTg-IgA result during patient evaluation would prompt the gastroscopy, reclassifying it from low-risk dyspepsia. A finding of Grade A esophagitis was not considered a clinically actionable finding.

Healthcare utilization and outcomes in the year following each gastroscopy were obtained from the National Ambulatory Care Reporting System and Discharge Abstract databases. Follow-up appointments with gastroenterologists who completed the gastroscopies and primary care visits related to dyspepsia or the upper gastrointestinal tract were obtained from physician billing claims data.

Data collected from chart reviews were anonymized and recorded in an electronic database. Descriptive statistics were reported as counts and proportions for categorical variables and median and interquartile ranges for continuous variables. Counts and proportions of low-risk endoscopies and corresponding clinically actionable findings were stratified by age <65, <60, and <55.

This study was approved by the University of Alberta Research Ethics Board. Given the retrospective non-interventional nature of the study, informed consent was waived. This study was performed in collaboration with the University of Alberta Physician Learning Program.

## Results

**Figure 1.**
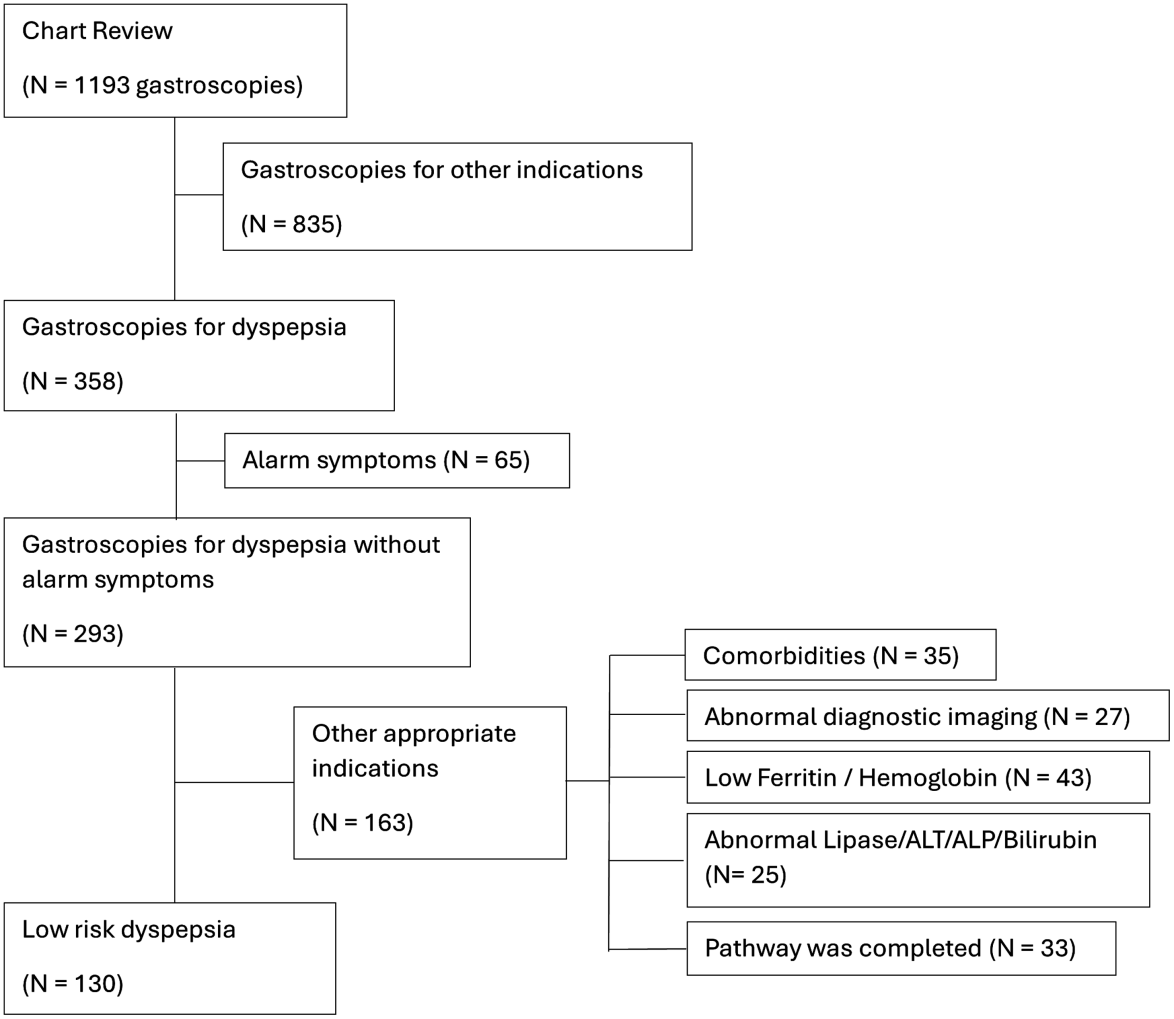
Flow chart for patient gastroscopies for inclusion and exclusion.

Out of the 1241 outpatient gastroscopy charts sampled on patients aged 18–65 years, 2 were duplicates and 46 were blank leaving a final sample size of 1193. Of the 1193 charts reviewed, 358 (30.0%) met our inclusion criteria for the indication of dyspepsia and 65/358 (18.2%) had alarm symptoms. Among the remaining 293/358 gastroscopies performed for dyspepsia without alarm symptoms, 163/293 (55.6%) had other appropriate indications such as abnormal laboratory testing, abnormal diagnostic imaging results, other comorbidities, and/or evidence of dyspepsia pathway adherence (see Figure 1).

Overall, 130/358 (36.0%) of the gastroscopies were performed for low-risk dyspepsia. That is, they were <65 years old and exhibited no alarm symptoms or documented appropriate indications for the gastroscopy. Gastroscopies labelled as dyspepsia and “Diagnostic-other” accounted for 82/130 (63.1%) and 14/130 (10.8%), respectively. The second chart review made up 34/130 (26.1%) of low-risk gastroscopies.

Among the 130 low-risk gastroscopies, 121 (93.1%) had a biopsy taken, but we did not document their location (esophagus, stomach, and duodenum) In 9 (6.9%) patients there were clinically significant findings (Table 1). See Table 1 for ages. The most common clinically significant finding was eosinophilic esophagitis (3/130, 2.3%). In one patient a benign gastric ulcer was found, and in another, a small gastric adenomatous polyp was found and removed. A follow-up gastroscopy for the polyp was normal. There were 14/130 (10.8%) patients with clinically significant findings that could have been diagnosed and treated non-invasively including 13 with *Helicobacter pylori* infection and one with celiac disease. There were no findings of Grade >B esophagitis in any of the patients. Grade A esophagitis was not considered a clinically important finding. 10/130 low-risk patients had LA Grade A esophagitis, 5 were prescribed PPIs within the year prior, and 5 were not prescribed PPIs.

**Table 1.** Gastroscopy/biopsy findings among low-risk dyspepsia.

Endoscopy findings (age)	*n* (%) (*N* = 130)
**Clinically actionable findings that could only be found through** **gastroscopy**	**9 (** **6.9)**
Two gastric ulcers, benign (50 y)	1
Candida esophagitis (62 y)	1
Eosinophilic esophagitis (30 y, 36 y, 43 y)	3
Gastric adenoma, no high-grade dysplasia (46 y)	1
False negative *H. pylori* (54 y)	1
Pill esophagitis (22 y)	1
Eosinophilic gastritis (56 y)	1
**Other** **findings**	**14 (** **10.8)**
*H. pylori* (no prior testing for Hp or antibiotics) (36–62 y)	11
Celiac disease (no prior tTg-IgA test) (29 y)	1
Untreated *H. pylori* (positive urea breath test, no antibiotics) (32 y, 65 y)	2
**No** **rmal**	**107** **(82.3)**

Of the 130 low-risk gastroscopies, 80 (62.0%) were booked direct-to-procedure without a prior separate gastroenterologist clinic-based consultation. Among those with evidence of pathway adherence, 2/33 (6.1%) had clinically significant findings: Candida esophagitis (61-year-old) and celiac disease (51-year-old).

Table 2 shows healthcare utilization during the post-endoscopy year. No patients were hospitalized related to dyspepsia during this period. One patient made 3 emergency department/urgent care visits, having a diagnosis of esophagitis and unspecified abdominal pain. Among the low-risk cohort, 35/130 (27.0%) had at least one follow-up appointment with the gastroenterologist who performed the gastroscopy. A total of 29 patients visited their primary care physician in relation to upper gastrointestinal tract concerns including dyspepsia. No upper gastrointestinal cancers were diagnosed during the follow-up period.

**Table 2:** Healthcare utilizations in the year following for low-risk dyspepsia patients.

Healthcare utilizations	*N* (*N* = 130)
Emergency department/urgent care visits*	
For dyspepsia	1
Diagnosis	Unspecified upper abdominal pain
For upper gastrointestinal symptoms	2
Diagnosis	Esophagitis; unspecified gastritis
Hospitalizations	0
General practitioner visits related to upper gastrointestinal symptoms	29
Median (IQR, interquartile range) days from gastroscopy to first general practitioner visit for upper gastrointestinal symptoms	35 (17–93)
General practitioner visits related to dyspepsia	9
Median (IQR, interquartile range) days from gastroscopy to first GP visit for Dyspepsia	82 (14–112)
Gastroenterologist follow-up	35
Median (IQR, interquartile range) days to gastroenterologist follow-up	61 days (27–110)
Additional gastroscopy	0
Previous gastroscopy prior to gastroscopy for dyspepsia	36
Gastrointestinal cancer diagnosis within 1 year following EGD	0

^*^Emergency department/urgent care visits were made by one individual.

Table 3 shows adherence to pathway recommendations prior to endoscopy among the low-risk patients. The use of testing to diagnose *Helicobacter pylori* was low at 14.6% (19/130). Documentation of proton pump inhibitor use was low at 63% (82/130).

**Table 3.** Pathway components completed among low risk dyspepsia.

Primary care pathway components	Low risk dyspepsia patients (*N* = 130) *n* (%)
Any blood work	106 (81.5)
Any diagnostic imaging (ultrasound, computerized tomography, X-ray with or without fluoroscopy of the abdomen, pelvis, or small bowel)	28 (21.1)
Urea breath test	19 (14.6)
Any proton pump inhibitor	82 (63.0)
Domperidone or metoclopramide	7 (5.4)
Any histamine H2-receptor antagonists	4 (3.1)

Table 4 shows the effect of different age cutoffs on low-risk dyspepsia gastroscopy counts and the yield of clinically actionable findings. By decreasing the age threshold there is little change in the proportion of low yield procedures. The percentages of actionable findings and findings that could have been found non-invasively remained similar among the 3 age groups.

**Table 4.** Comparison of gastroscopy indications dyspepsia with different age cut-offs.

	Age <65	Age <60	Age <55
	*n* (%)	*n* (%)	*n* (%)
EGDs* for dyspepsia	358	304	255
EGDs for dyspepsia with alarm symptoms	65 (18.2)	57 (18.8)	49 (19.2)
EGDs for dyspepsia with other appropriate indications	130 (36.3)	108 (43.7)	91 (44.2)
EGDs where pathway appropriately followed	33 (20.2)	27 (19.4)	23 (20.0)
EGDs for low-risk dyspepsia	130 (36.3)	112 (36.8%)	92 (36.0)
Clinically actionable findings	9 (7%)	7 (6.3)	6 (6.5)
Findings could have been found non-invasively	14 (10.7)	13 (11.6)	9 (9.9)

^*^EGDs = esophagogastroduodenoscopy.

## Discussion

The 36% observed total (130/358) of low-risk gastroscopies in our study exceeds the ASGE and CAG guideline recommendation that the proportion of gastroscopies lacking an appropriate indication should be below 20% of all performed gastroscopies.^19,20^ The rationale for conducting gastroscopy in cases of low-risk dyspepsia is often unclear, even upon thorough chart review.

In our cohort, clinically actionable findings occurred in 6.9% (9/130) of patients, which was higher than a study conducted in Calgary, Alberta where only 2.1% of patients had clinically actionable findings using similar criteria.^30^ The proportion of low-risk gastroscopies was similar between the 2 studies (36% and 35%, respectively), and in both cohorts, no malignancies were identified in the year following, suggesting consistency across the province.

The findings of eosinophilic esophagitis (3/130) were unexpected, as this condition is usually diagnosed in patients with dysphagia, which is an alarming symptom and an accepted indication for gastroscopy.

Among the 130 low-risk gastroscopies, biopsies were taken in 121 cases but we did not document from where they were obtained. Therefore, it is possible that for some diagnoses, for example, *H. pylori* gastritis or eosinophilic esophagitis, the frequency may be underestimated. Had biopsies been taken in all 130 procedures, it is possible that the number of clinically significant findings would have increased. A finding of Grade A esophagitis was not considered a clinically actionable finding, as was done in a similar study conducted in Calgary.^30^ According to the Lyon 2.0 consensus, LA-A esophagitis is inconclusive evidence for GERD, both on and off PPI therapy.^31^

A total of 10/130 low-risk patients had LA Grade A esophagitis, 5 of whom had been prescribed PPIs within the year prior and 5 who were not. It is possible that the use of PPI either completely healed or masked more severe esophagitis. There were no cases of Grade >B esophagitis in any of the patients. Our cohort reflects the current EZ practice of performing gastroscopy in low-risk dyspepsia patients including the frequency with which biopsies are taken during the procedure.

Our dyspepsia definition is inclusive, accommodating potential overlaps with other conditions and encompassing patients with organic symptom causes. Our sample includes the full range of dyspepsia cases and reflects individuals with upper GI symptoms who are eligible for our Alberta dyspepsia pathway.^27^ While acknowledging dyspepsia and GERD overlap, Alberta employs a distinct GERD pathway centered on predominant heartburn and/or regurgitation symptoms.^26^ It is acknowledged that there is significant overlap between dyspepsia and GERD and that a symptom-based diagnosis of GERD has limited sensitivity and specificity.^9,32,33^

In the DIAMOND study, the sensitivity and specificity for a symptom-based GERD diagnosis was limited at 63 and 63% by family physicians and 67 and 70% by gastroenterologists.^32^ In practice in GERD, dyspepsia and patients with overlapping symptoms treatment with a PPI is usually tried first.

The incidence of gastric and esophageal cancer in Canada is low. In males, 2.4% of all cancers in Canada are gastric and 1.6% are esophageal; in females, the rates of these cancers are 1.4% and 0.5%, respectively. These cancers primarily affect those aged 60 and above.^34,35^ Given our small sample size and ages <65, our absence of cancer findings in the following year is not surprising. Notably, Choosing Wisely Canada changed its recommended age threshold from 65 to 60 during the time our study was conducted. Our data supports the low upper GI cancer risk in this age group. However, the small sample size of our study makes it impossible to draw a reliable conclusion on the impact of changing the age threshold on cancer detection. Furthermore, our small sample and short follow-up of 12 months prevent definitive comments on missed upper GI cancers. Changing the age cutoff (<65, <60, and <55) did not change the proportion of clinically actionable findings.

There are long wait times in Canada to access gastroenterology services, especially for low-risk conditions,^21^ making the overuse of gastroscopy to investigate symptoms of dyspepsia an important issue to address.^35^ Clinical care pathways have been developed in Alberta to optimize the care of common low-risk gastrointestinal conditions within primary care.^24–27^ For dyspepsia, this includes a test and treat strategy for *Helicobacter pylori* and a trial of proton pump inhibitors.^2,5,16,17,28,29,36,37^ Despite widespread recommendations supporting this approach, evidence for pathway adherence in our cohort was low. Only 20% completed the main treatment components of the pathway prior to gastroscopy. Testing for *Helicobacter pylori* was low at 14.6%, possibly reflecting low awareness that *H. pylori* can play a role in dyspepsia and the burden of urea breath testing for patients. After this study, stool antigen testing was introduced which may increase the ease of *H. pylori* testing and, therefore, pathway adherence. Evidence of proton pump inhibitor trial prior to endoscopy, another key component of the dyspepsia pathway, was low at 63%.

The performance of low-value diagnostic tests to rule out underlying disease and reassure patients is not unique to dyspepsia and is a persistent problem.^38^ Reasons for unnecessary gastroscopy referral and performance include: low awareness of dyspepsia guidelines, complexity and overlap of symptoms and causes, failure to respond to therapy, unfiltered open-access referral systems, physician remuneration, patient/referring practitioner expectations, a desire for self and patient reassurance there is no serious cause, and a perception that gastroscopy performance might save costs and prevent emergency room visits, particularly in highly health-anxious patients.^35,39–42^ To reduce low-value gastroscopy in dyspeptic patients, strategies like web-based patient education, physician audit and feedback, nurse-led shared appointments, referral filters, guideline adaptation and implementation, and decision-aids have been tried with varying success.^35,43,44^ In Alberta, we do not have open-access gastroscopies. We have direct-to-procedure (DTP) bookings combining the consultation and gastroscopy at the same time. The decision for DTP procedures is made by the physician triaging the referral. Effective interventions likely require a combination of targeting physicians, patients, and the health-system.

As mentioned, audit and feedback is a successful approach to reducing low-value procedures that generates reflective discussion with physicians about root causes and solutions.^45,46^ An audit and feedback intervention in Calgary, Alberta demonstrated a 35 to 22% reduction in low-risk gastroscopies over one year (K Novak unpublished data). Similar audit and feedback sessions based on these results were developed by the Physician Learning Program and delivered to participating gastroenterologists in the Edmonton Zone. During these sessions, gastroenterologists identified barriers and facilitators to reducing gastroscopies for low-risk dyspepsia and brainstormed solutions that they committed to implementing in their practice. A follow-up and evaluation of gastroenterologist’s commitments are planned to observe if there was a change in their gastroscopy practices.

Limitations to this study included data availability and validity. A large proportion of gastroscopy reports exhibited inconsistent or unclear descriptions of the procedure indication and either did not match the indication code in the hospital service logs or were generically labelled as being performed for the reason “other” necessitating labor-intensive manual review to determine if the procedure was performed for dyspepsia. For example, many reports exhibited more than one indication, in addition to dyspepsia. Thus, we may have underestimated the number of procedures performed for low-risk dyspepsia. Another challenge with the procedure reports is that not all pre-existing conditions are captured. A patient may have other conditions that would explain upper abdominal symptoms despite having a normal gastroscopy, such as biliary colic. When symptoms are suggestive of possible biliary colic, for example, attacks of pain in the right upper quadrant of the abdomen, an abdominal ultrasound should be considered. However, biliary colic is not part of our dyspepsia definition. Our dyspepsia definition and pathway also did not include routine testing for gastroparesis. Another limitation is we did not collect data on other potential contributing factors, such as duration of symptoms, mental health comorbidities, or personal stress that may predict low-risk dyspepsia. It should be acknowledged our chart review identified inconsistencies in using “dyspepsia” as a procedure code. We also did not collect data on physician identifiers. We therefore did not analyze differences in outcomes among individual physicians, whether they were academic physicians paid by an alternative funding plan or fee-for-service, nor between hospital sites. Challenges with using administrative data to assess clinical practice and quality are not new and this study presents another example of these challenges.^47^

The COVID-19 pandemic had profound effects on healthcare utilization during this period, thus our data may not accurately reflect pre-pandemic conditions.^48^ During the study period, the dyspepsia pathway was not fully implemented in the Edmonton zone, which may have affected pathway adherence data. This study focused on a cohort of low-risk patients who received gastroscopy, not on adherence to the pathway for patients managed in primary care. Another limitation was changing Choosing Wisely age thresholds. In 2019, Choosing Wisely Canada used age 65 as the cut-off for gastroscopy for patients with dyspepsia. This changed in 2021 to be <60.^15^ To address this limitation, we stratified our results by age cut-offs of 55, 60, and 65 years.

## Conclusion

In Alberta, the proportion of gastroscopies performed for low-risk dyspepsia is higher than recommended by current practice standards. These results suggest further improvements in awareness, education, and system-level transformation are needed to reduce low-yield gastroscopy in patients with this common symptom complex. Further efforts should be made to decrease the use of gastroscopy among patients who can be safely managed in their primary care medical home without a referral by encouraging consideration of, and adherence to, the dyspepsia primary care pathway at, and prior to, the time of gastroscopy performance decisions. Interventions targeting physicians, patients, and the healthcare system are most likely needed. A decrease in the number of low-risk dyspepsia gastroscopies will result in health system savings and increase endoscopy access. Ongoing efforts to understand this practice gap and promote practice change are focused on improving health system performance.

## Supplementary Material

gwae003_suppl_Supplementary_Materails

## Data Availability

The data underlying this article cannot be shared publicly due to patient confidentiality.
